# Supplementary material for: A bimetallic nanoplatform for STING activation and CRISPR/Cas mediated depletion of the methionine transporter in cancer cells restores anti-tumor immune responses
Source: Nat Commun. 2023 Aug 2;14:4647. doi: 10.1038/s41467-023-40345-3 (PMC10397352; doi:10.1038/s41467-023-40345-3)
Supplement: Supplementary file 1 — Supplementary Information [file 41467_2023_40345_MOESM1_ESM.pdf]

Supporting Information for

A bimetallic nanoplatform for STING activation and CRISPR/Cas mediated depletion of the methionine transporter in cancer cells restores anti-tumor immune responses

Ying Huang,[1,2] *Geng Qin,**[1,2] *TingTing Cui,*[1,2] *Chuanqi Zhao,**[1,2] *Jinsong Ren,* [1,2] *and Xiaogang Qu**[1,2]

AUTHOR ADDRESS

[1] Laboratory of Chemical Biology and State Key Laboratory of Rare Earth Resource Utilization, Changchun Institute of Applied Chemistry, Chinese Academy of Sciences, Changchun, Jilin 130022, P. R. China

[2] School of Applied Chemistry and Engineering, University of Science and Technology of China, Hefei, Anhui 230026, P. R. China

*email: xqu@ciac.ac.cn ; zhaocq@ciac.ac.cn; qingeng@ciac.ac.cn

Supplementary Methods

Reagents and Materials:

Zinc nitrate hexahydrate (Zn $(NO_3)_2·6H_2O$, 99.0%) was purchased from Shanghai Chemical Reagent Company (Shanghai, China); 2-methylimidazole was purchased from Sigma-Aldrich; Manganese (II) acetyllacetonate (96%) was purchased from Alfa Aesar Company; 3-(4, 5-dimethyl-2-thiazolyl)-2, 5-diphenyl-2-Htetrazolium bromide (MTT) was from Sangon Biotechnology Inc. (Shanghai, P.R. China). Enzyme linked immunosorbent assay (ELISA) kits were purchased from Multi Sciences (Lianke) Biotechnology Co., Ltd (Beijing, China). Cell staining buffer and antibody were purchased from Biolegend. 75 μm nylon cell strainer and 0.4 μm-sized Transwell®plates were purchased from Corning. JC-1 was purchased from Solarbio. Animal Tissue Cell Total DNA Extraction Kit was purchased from Shenzhen Dakewe Bio-engineering Co.,Ltd. All primers were purchased from Kumei Bio. All other reagents were analytical grade and used as received. RPMI-1640 (without methionine) was purchased from Procell. pLV3-U6-Sting(mouse)-sgRNA2-Cas9-EGFP-Puro was purchased from miaolingbio. Ultrapure water (18.2 MΩ;Millpore Co. USA) was used throughout the experiment.

Measurements and Characterizations:

Scanning electron microscopic (SEM) images were carried out using a Hitachi S-4800 Instrument (Japan). Transmission electron microscopic (TEM) images and high-angle annular dark-field scanning TEM (HAADF-STEM) images were captured by a FEI TECNAI G2 20 high-resolution transmission electron microscope operating at 200 kV. The zeta-potential of the nanoparticles was measured in a Zetasizer 3000HS analyzer.

The FT-IR characterization was recorded on a BRUKE Vertex 70 FT-IR spectrometer. The elemental analysis was performed by an ICAP 6000 ICP-OES system (Thermo Scientific, Waltham, MA, USA). The X-ray photoelectron Spectroscopy (XPS) spectra were analyzed by Thermo Fisher Scientific ESCALAB 250Xi Spectrometer Electron Spectroscopy (America). The UV-Vis absorption measurement was recorded using a JASCO V550 UV-Visible spectrophotometer (JASCO International Co., LTD., Tokyo, Japan). The High Performance Liquid Chromatography (HPLC) was measured by Ultimate 3000. The confocal laser scanning microscopy (CLSM) characterization was acquired by a (Nikon Eclipse Ni-E, Japan) top-of- the-line motorized upright. The flow cytometry data was obtained by BD LSRFortessa™ Cell Analyzer. T1-weighted MR images were acquired using Siemens Prisma 3.0 T MR scanner (Erlangen, Germany) with gradient strength up to 80 Mt m-1. Imaging parameters were as follows: repetition time (TR), 12000ms; seven inversion recovery times (TI = 20, 40, 80, 160, 320, 640, 1280 and 2560 ms).

Antibody	Host species	Dilution	Duration	Supplier	Conjugation
β-actin	Mouse/Rabbit	1:1000	12h	Bioss	N/A
TBK1	Rabbit	1:1000	12 h	Beyotime	N/A
P-TBK1	Rabbit	1:1000	12 h	Beyotime	N/A
IFN-Beta	Rabbit	1:1000/1:500	12 h	Bioss	N/A

SLC43A2	Rabbit	1:1000	12 h	Bioss	N/A
TMEM173/STING	Rabbit	1:500	12 h	Proteintech	N/A
CD3	Mouse	1:200	12h	Biolegend	N/A
CD28	Mouse	1:200	12h	Biolegend	N/A
CD11c	Mouse	1:16	30 min	Biolegend	APC
CD80	Mouse	1:30	30 min	Biolegend	FITC
CD86	Mouse	1:30	30 min	Biolegend	PE
CD8	Mouse	1:16	30 min	Biolegend	PE
CD3	Mouse	1:50	30 min	Biolegend	FITC
CD4	Mouse	1:16	30 min	Biolegend	APC
CD8	Mouse	1:16	30 min	Biolegend	APC
CD45	Mouse	1:16	30 min	Biolegend	perCP/Cyanine 5.5
F4/80	Mouse	1:16	30 min	Biolegend	FITC
CD206	Mouse	1:16	30 min	Biolegend	perCP/Cyanine 5.5
anti Mouse/Rabbit IgG(H+L)	Goat	1:500	30 min	Bioss	HRP
anti Rabbit IgG (H+L)	Goat	1:500	30 min	Beyotime	FITC

Supplementary Table 1. Antibodies used for this work.

**gRNA and Primers used for RT-qPCR**	
**Gene name**	**Sequence(5’-3’)**
IFN-beta-F	TCCTGCTGTGCTTCTCCACCACA
IFN-beta-R	AAGTCCGCCCTGTAGGTGAGGTT
Cxc110-F	ATCATCCCTGCGAGCCTATCCT
Cxc110-R	GACCTTTTTTGGCTAAACGCTTTC
mtDNA-loop sense	TCACCCTATTAACCACTCA
mtDNA-loop anti scense	AGACAGATACTGCGACATA
MT-ND1 sense	CACCCAAGAACAGGGTTTGT
MT-ND1 anti-sense	TGGCCATGGGTATGTTGTTAA
SLC43A2-F	CAGCATCCTTGAGTTCCTGGTC
SLC43A2-R	TGATGTAGCCGATGACAGGAGC
βActin-F	CATTGCTGACAGGATGCAGAAGG
βActin-R	TGCTGGAAGGTGGACAGTGAGG
Non-targeting gRNA 1F	CACCGCTGAAAAAGGAAGGAGTTGA
Non-targeting gRNA 1R	AAACTCAACTCCTTCCTTTTTCAGC

Supplementary Table 2. Primers used for RT-qPCR and gRNA.

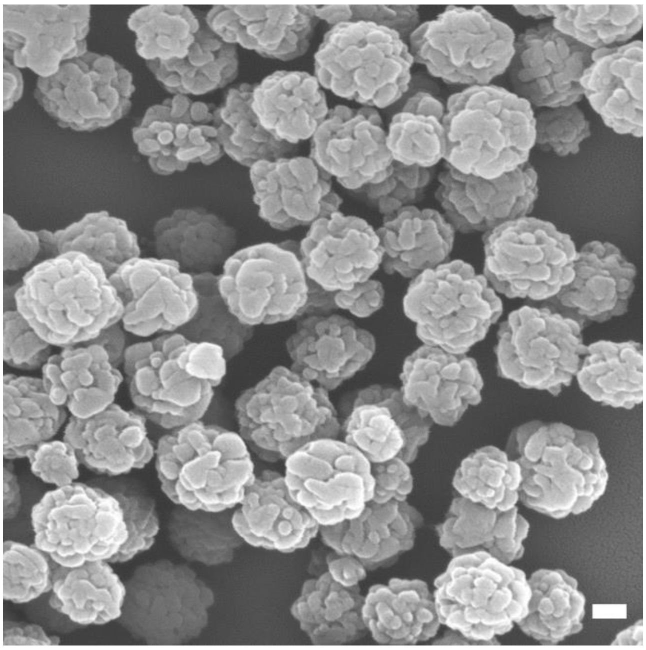

Supplementary Figure 1. SEM images of PMZ.(The scales is 100 nm.) Representative results showed in Figure were obtained from three independent samples.

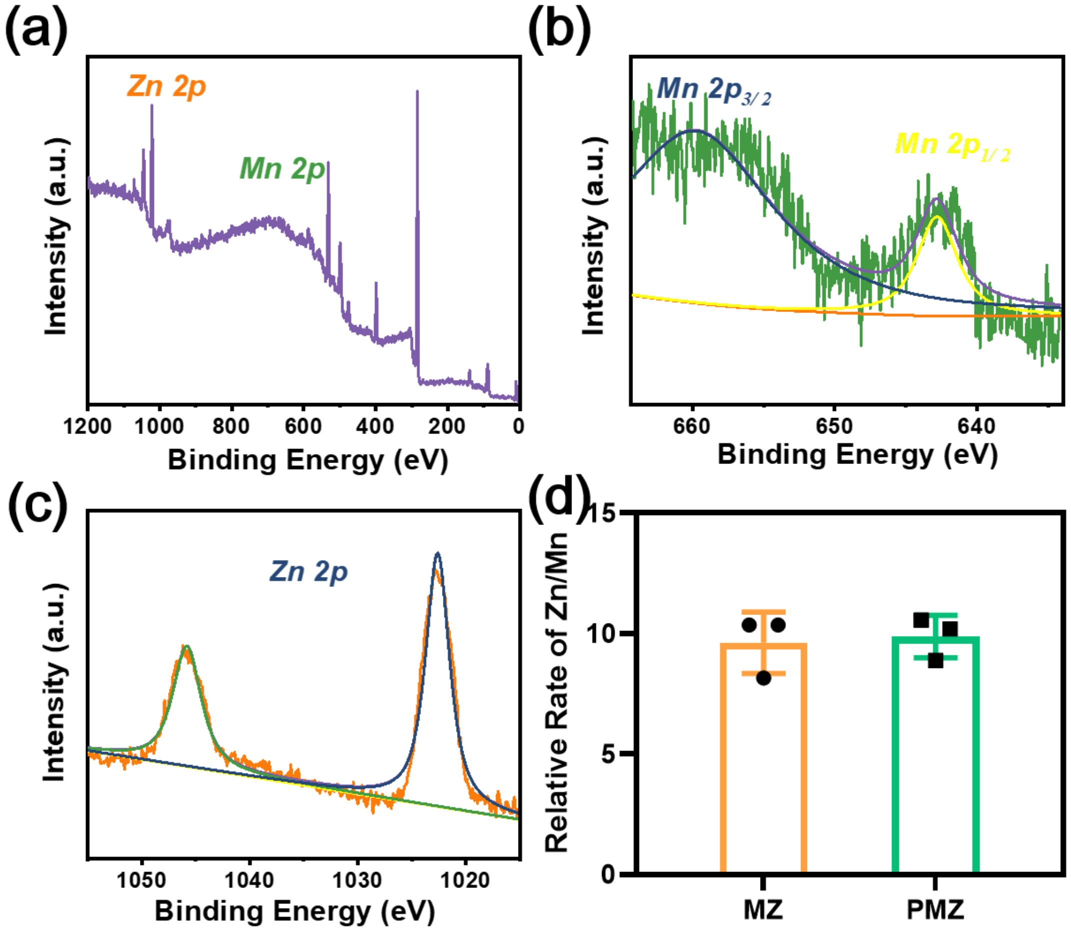

Supplementary Figure 2. (a) XPS spectra of MZ. XPS high-resolution scans of (b) *Mn 2p* and (c) *Zn 2p* peaks in MZ. (d) ICP analysis of Zn/Mn ratio. Data were presented as mean ± SD ($n = 3$ independent experiments). Source data are provided as a Source Data file.

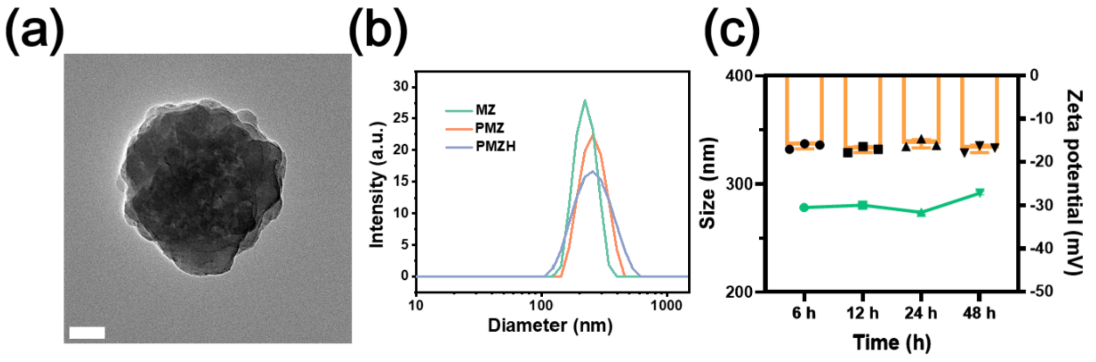

Supplementary Figure 3. (a) TEM of PMZH. Scale bar is 50 nm. (b) DLS profiles of PMZH (blue),PMZ (orange) and MZ (green) nanoparticles.PDI of PMZH is 0.236. Representative results showed in Figure a and b were obtained from three independent samples. (c) DLS/Zeta of PMZH after incubation in serum for different durations. Data were presented as mean ± SD ($n = 3$ independent experiments). Source data are provided as a Source Data file.

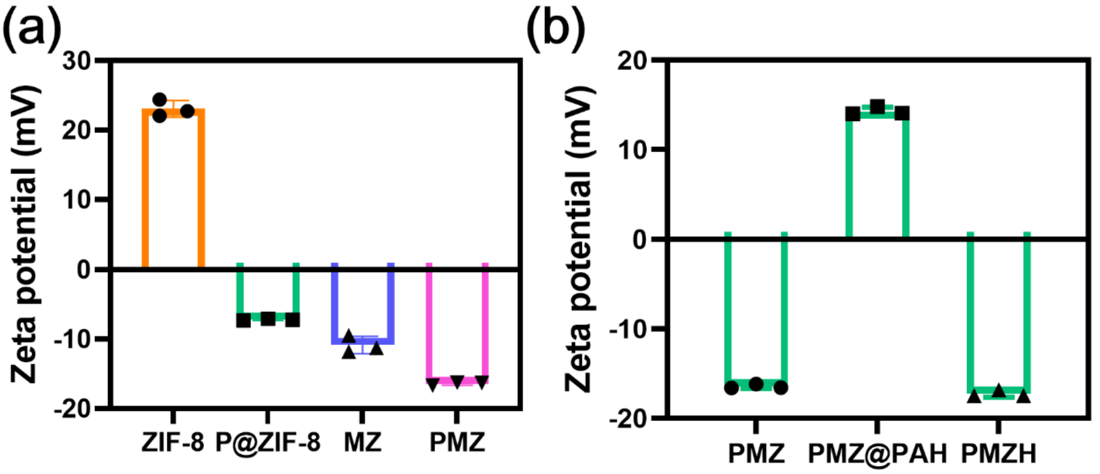

Supplementary Figure 4. (a) Zeta potention of ZIF-8, PZ, MZ, PMZ； (b) The Zeta potentials of the intermediate products during the preparation process of PMZH nanosystem. Data were presented as mean ± SD ($n = 3$ independent experiments in figure a and b). Source data are provided as a Source Data file.

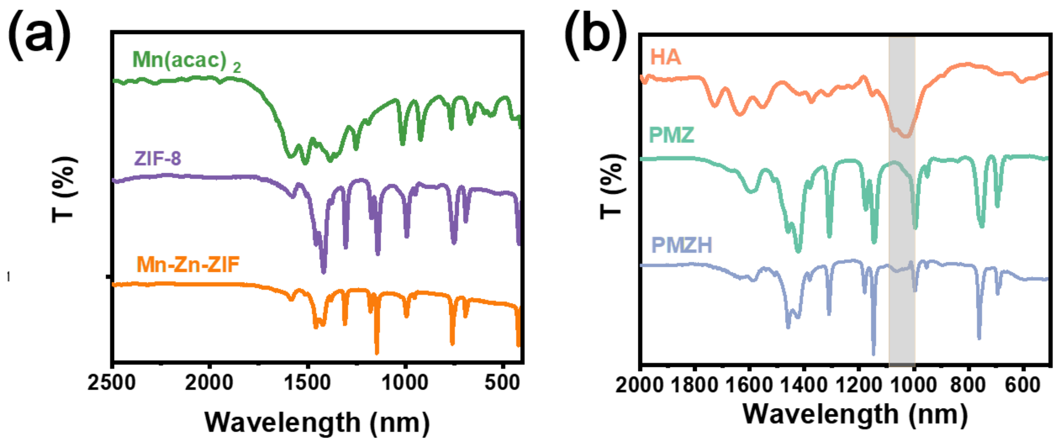

Supplementary Figure 5. (a) FT-IR spectra of $Mn(acac)_2$, ZIF-8, Mn-Zn-ZIF. (b) FT-IR spectra of HA, PMZ, and PMZH. Representative results showed in Figure a and b were obtained from three independent samples. Source data are provided as a Source Data file.

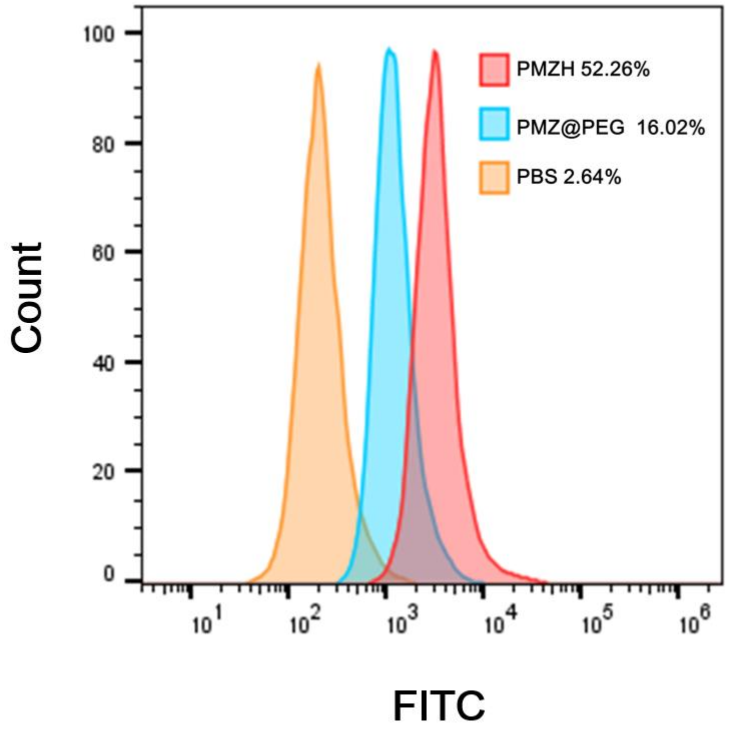

Supplementary Figure 6. Cellular uptake of FITC-modified PMZH and FITC-modified PMZ@PEG at 4 h. Representative results showed in Figure were obtained from three independent samples.

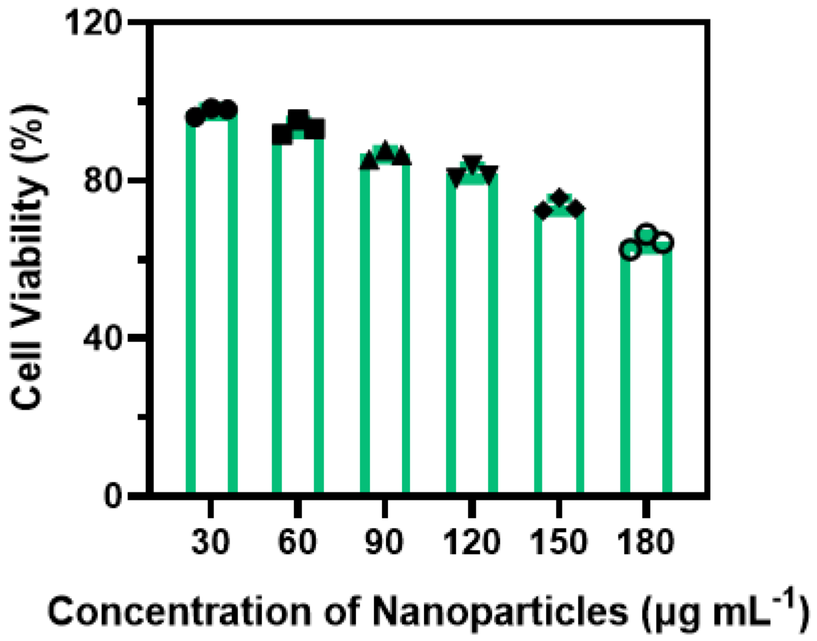

Supplementary Figure 7. Cell viability after different concentration of MZH nanoparticles for 12 h. Data were presented as mean ± SD (*n* = 3 independent experiments). Source data are provided as a Source Data file.

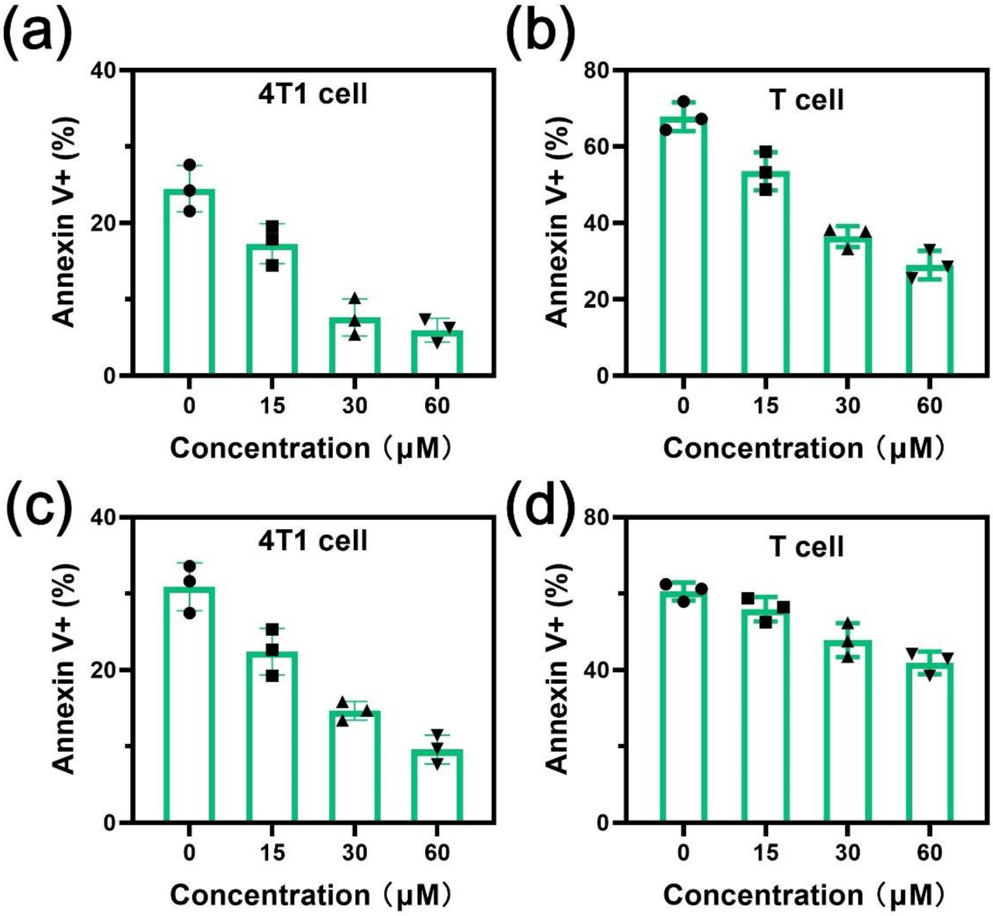

Supplementary Figure 8. Effect of methionine on viability of (a) 4T1 cells alone and (b) T cells alone. Effect of different concentrations of methionine on (c) 4T1 cells and (d) T cells viability when tumor and T cells compete for methionine in a Transwell system. Data were presented as mean ± SD (*n* = 3 independent experiments in figure a, b, c, d). Source data are provided as a Source Data file.

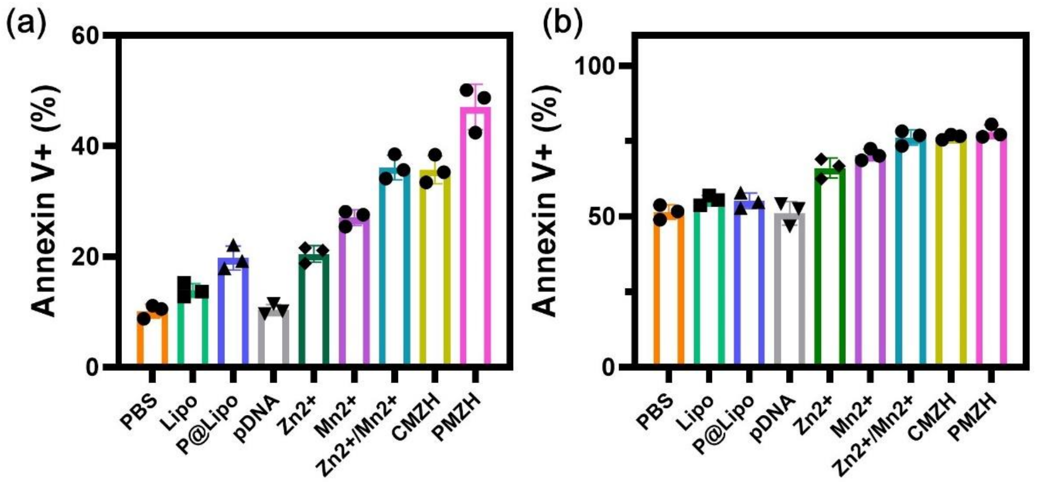

Supplementary Figure 9. In a non-Transwell system (a) Flow cytometry to detect the changes of

4T1 cell viability in different treatment groups. (b) Flow cytometry quantitatively assesses the changes in the viability of T cells in different treatment groups. Data were presented as mean ± SD ($n = 3$ independent experiments in figure a and b). Source data are provided as a Source Data file.

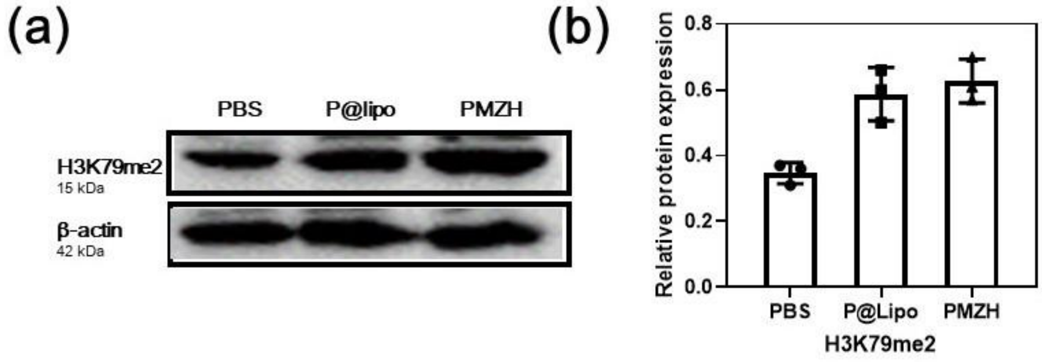

Supplementary Figure 10. Western blot (a) shows H3K79me2 in CD8 T^{+} cells. (b) Semi-quantitative analysis of H3K79me2 expression. Data were presented as mean ± SD ($n = 3$ independent experiments). Source data are provided as a Source Data file.

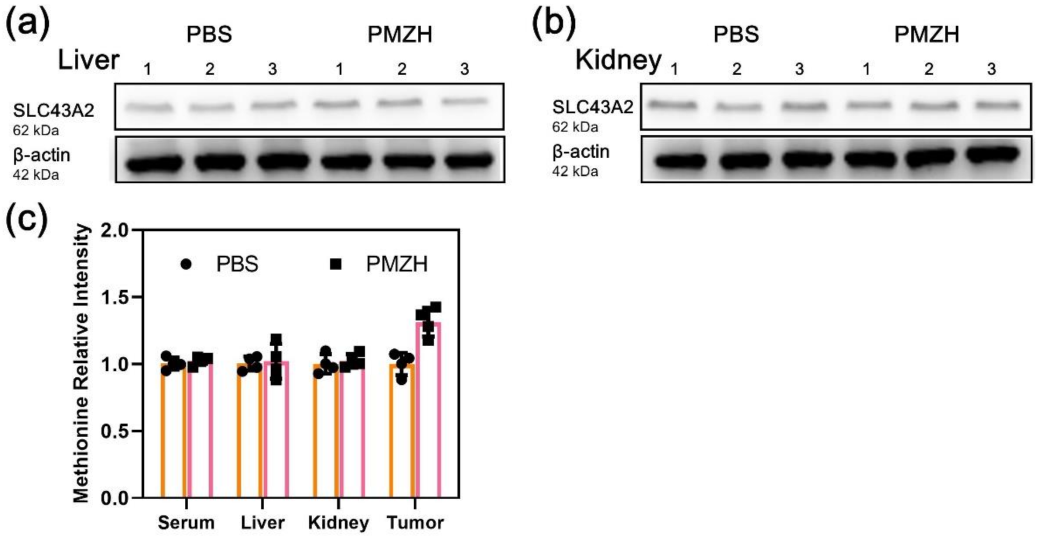

Supplementary Figure 11. Western blot analysis of SLC43A2 protein expression in (a) liver tissues, and (b) kidney tissues after indicated treatments. **(**c) Relative intensity of methionine. Data were presented as mean ± SD ($n = 4$ independent experiments). Source data are provided as a Source Data file.

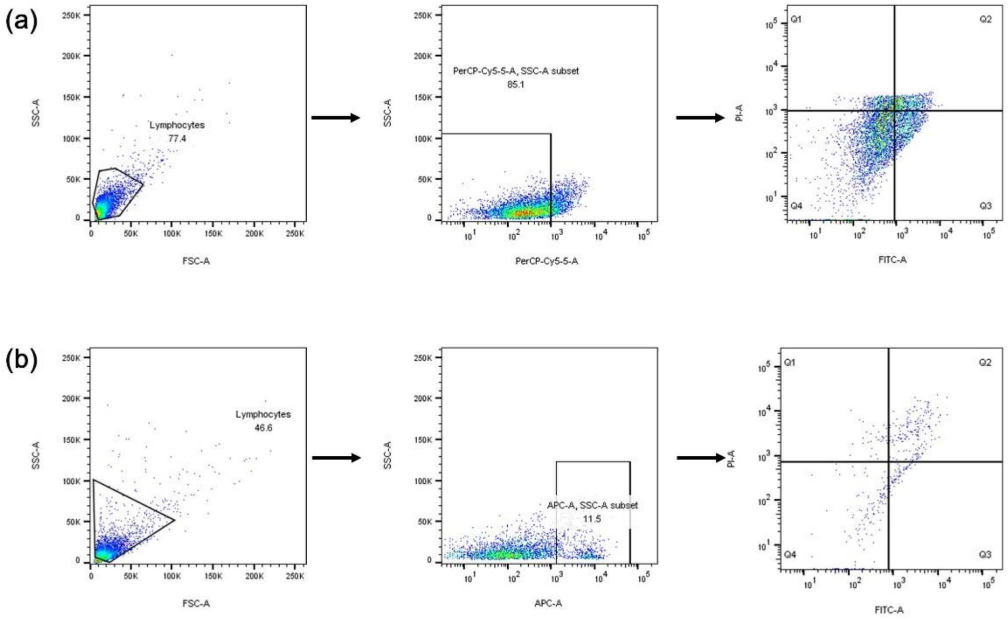

Supplementary Figure 12. Representative gating strategies for (a) CD45^{-} 4T1 cells apoptosis (Figure 4d, I, Supplementary Figure 8a, c, 9a) and (b) CD8^{+} T cells apoptosis (Figure 4c,j, Supplementary Figure 8b,d,9b.23a).

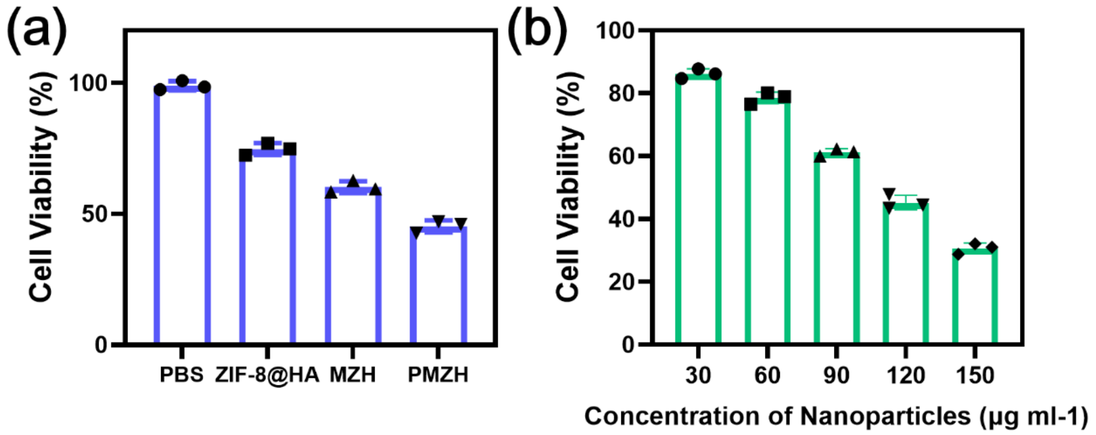

Supplementary Figure 13. (a) Cell viability after different treatments for 48h. (b) Cell viability after different concentration of PMZH nanoparticles for 48h. Data were presented as mean ± SD ($n = 3$ independent experiments in figure a and b). Source data are provided as a Source Data file.

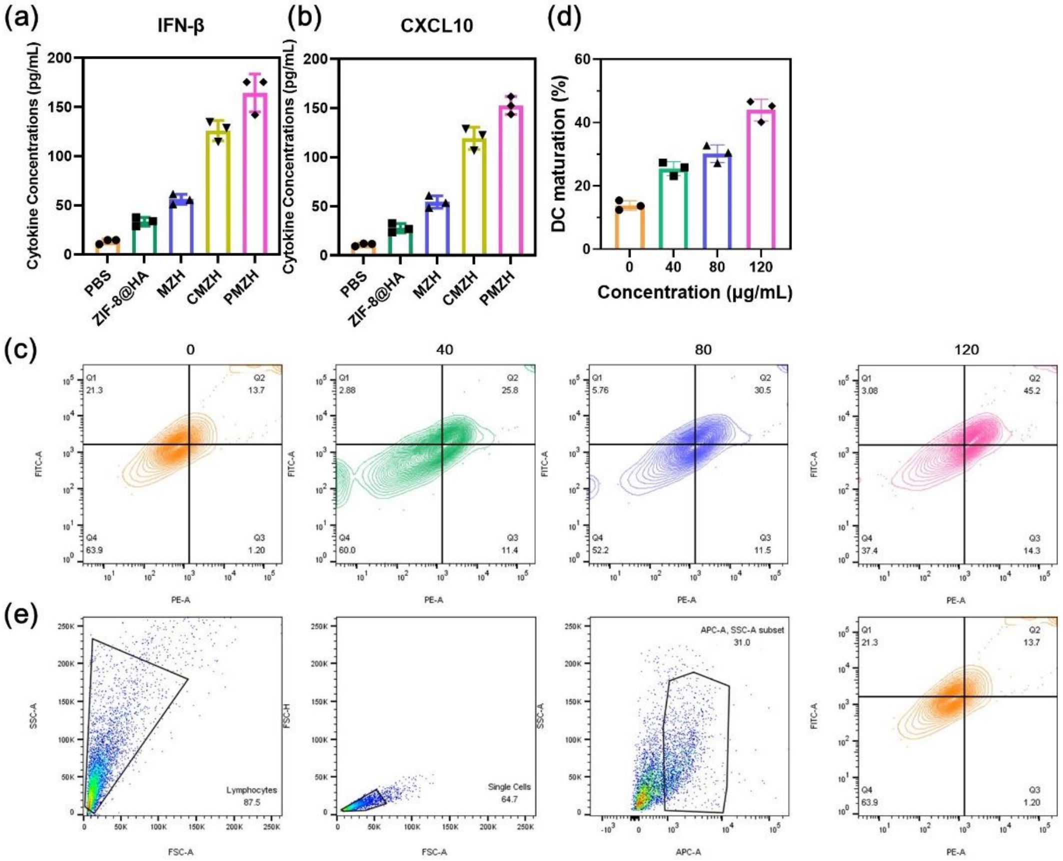

Supplementary Figure 14. The secretion levels of (a) IFN-β and (b) CXCL10 in the supernatant of BMDCs after different treatments. Representative (c) flow cytometric plots and (d) quantitative analysis of mature BMDCs ($CD80^{+}CD86^{+}$ in $CD11c^{+}$ cells) after incubation with various concentrations of PMZH. (e) Representative gating strategies for DC cells (Figure 7a, Supplementary Figure 14c, Supplementary Figure 19d). Data were presented as mean ± SD ($n = 3$ independent experiments in a ,b and d).Source data are provided as a Source Data file.

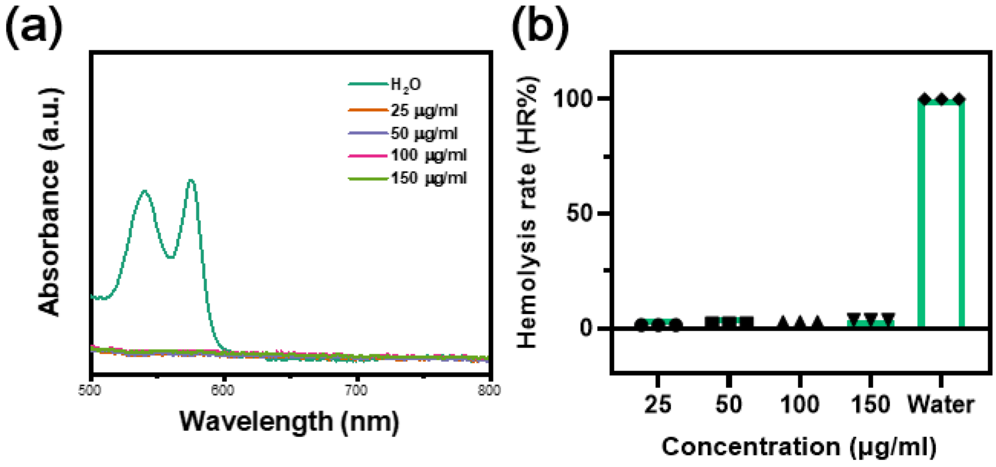

Supplementary Figure 15. a) Hemolysis test and b) Hemolysis rate (HR %) of PMZH. Data were presented as mean ± SD (n = 3 independent experiments).Source data are provided as a Source Data file.

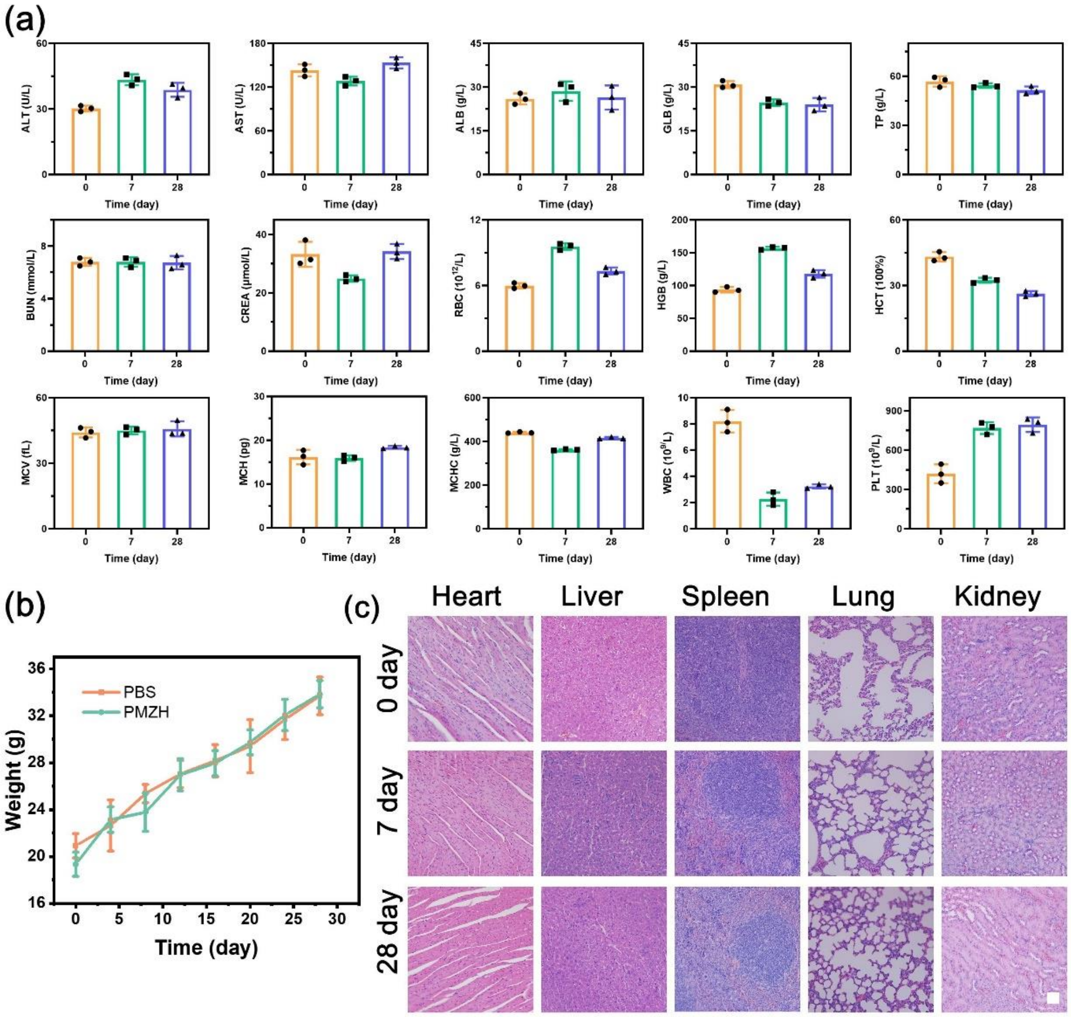

Supplementary Figure 16. In vivo toxicity. (a) Blood biochemical and hematological analysis of the healthy mice intravenously injected with PMZH at 7 and 28 days' post-injection. (b) Body weights of mice treated with PMZH, record every three days. Data were presented as mean ± SD (n = 3 mice per group in figure a and b.). (c) H&E images obtained from the major organs of PMZH-treated mice at 7 and 28 days' post-injection. n = 3 mice per group. Scale bars are 50 μm. Source data are provided as a Source Data file.

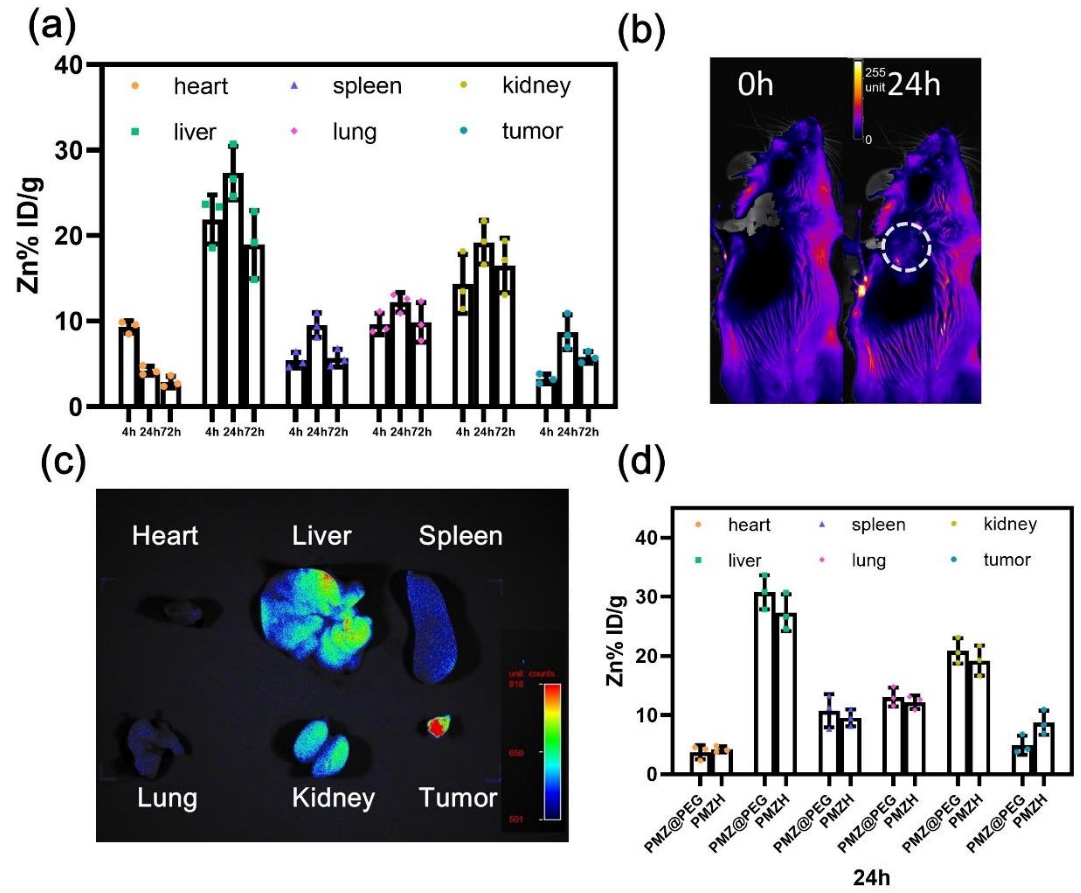

Supplementary Figure 17. (a) Biodistribution of Zn in major organs and tumors (ID% per tissue Zn) after intravenous injection of PMZH at different time intervals (4, 24, and 72 h). (b) In vivo fluorescence images of mice after intravenous injection 24h of DiD-labeled PMZH. (c) ex-vivo fluorescence images of mice after intravenous injection 24h of Cy5-labeled PMZH. (d) Biodistribution of Zn in major organs and tumors (ID% per tissue Zn) after intravenous injection of PMZ@PEG and PMZH at 24h. Data were presented as mean ± SD ($n = 3$ mice per group in figure a and d). Source data are provided as a Source Data file.

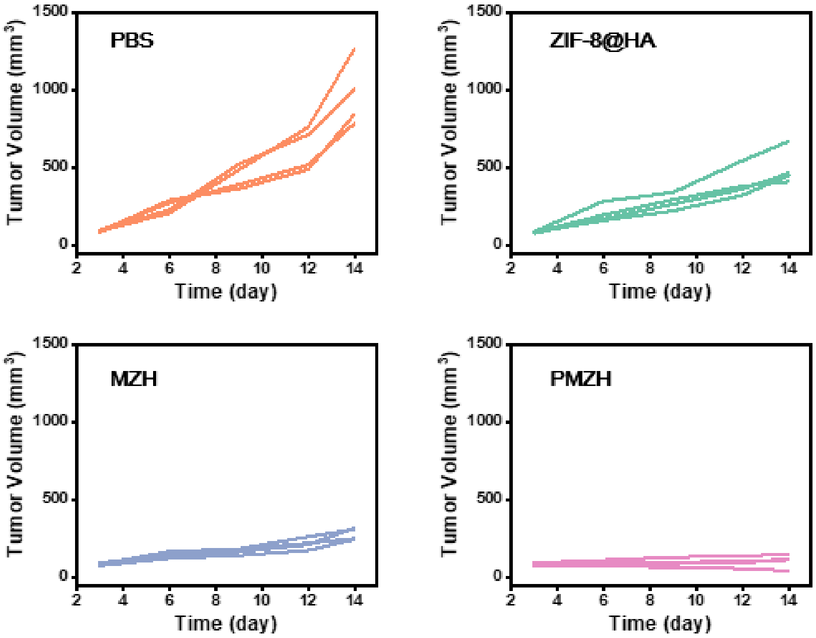

Supplementary Figure 18. Volume curves of tumor in PBS, ZIF-8@HA, MZH, and PMZH groups. *n* = 4 mice per group. Source data are provided as a Source Data file.

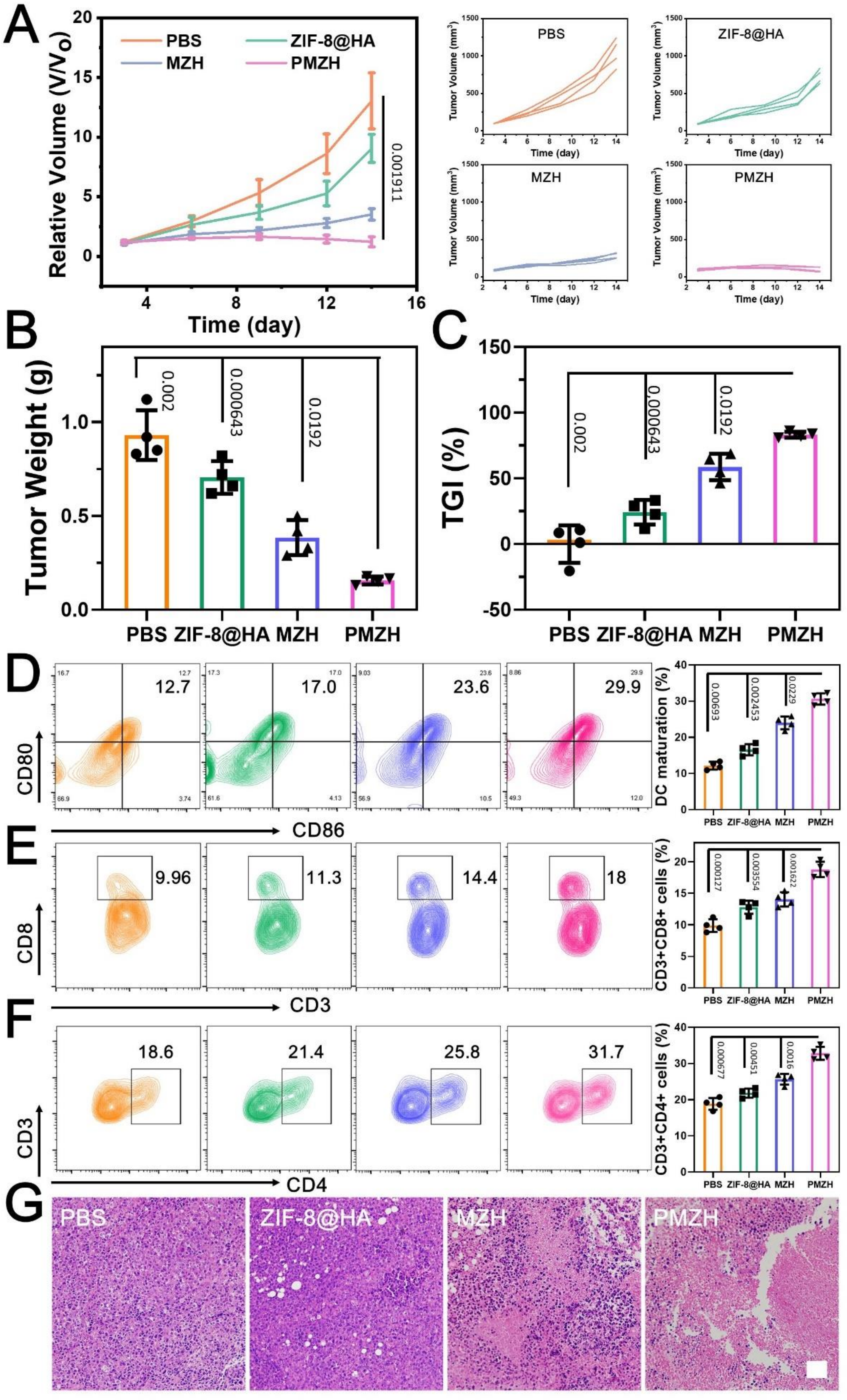

Supplementary Figure 19. Antitumor therapeutic effect of PMZH in CT26 tumor-bearing modle.

a) Tumor volume changes, b) tumor weights c) TGI rates after different treatments. d)Flow cytometry analysis of DCs (gating on CD11c+) infiltrated after treatment with different formulations. Populations of e) CD8+ and f) CD4+T cells in 4T1 tumor tissues after different treatment. g) H&E staining pictures of tumors after various treatments. (Scale bar: 50 μm) Data were presented as mean ± SD (n = 4 independent experiments in figure a-f). n = 3 mice per group in figure g. P values were assessed using Student's t test (two-tailed). Source data are provided as a Source Data file.

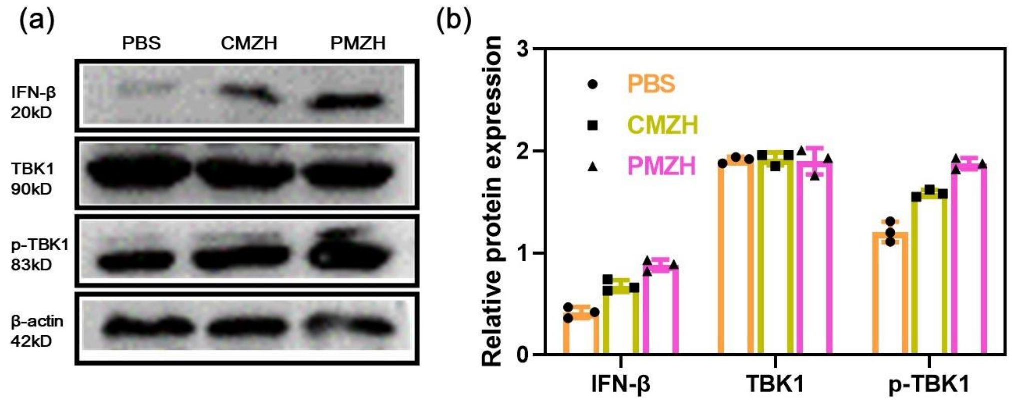

Supplementary Figure 20. a)Western blot analysis of STING pathway-related protein levels in tumor samples from different treatment groups. b) Relative protein expression. Data were presented as mean ± SD (n = 3 independent experiments per group). Source data are provided as a Source Data file.

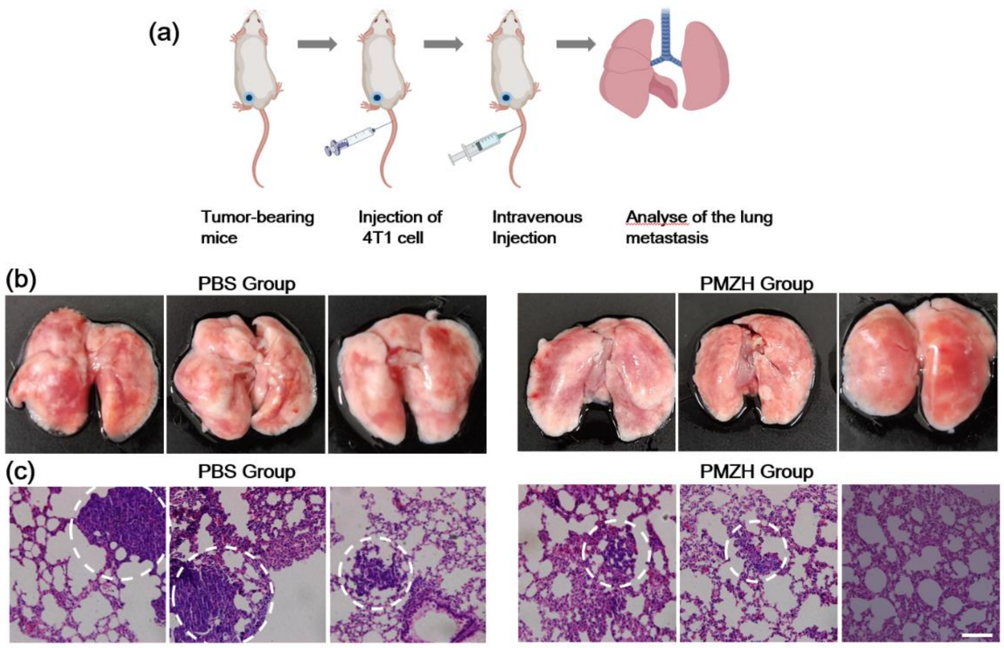

Supplementary Figure 21. Anti-metastasis effect of PMZH on mice with a 4T1 metastasis tumor model. (a) Schematic illustration of the experiment design to evaluate the lung metastases inhibition.(b) Representative photos of the sacrificed lung tissues. The white arrows indicate metastatic nodules. (c) The H&E staining of lung tissue section after various treatments. $n = 3$ mice per group.Scale bar: 100 μm. The black circles indicate the tumor in the lung tissue section.

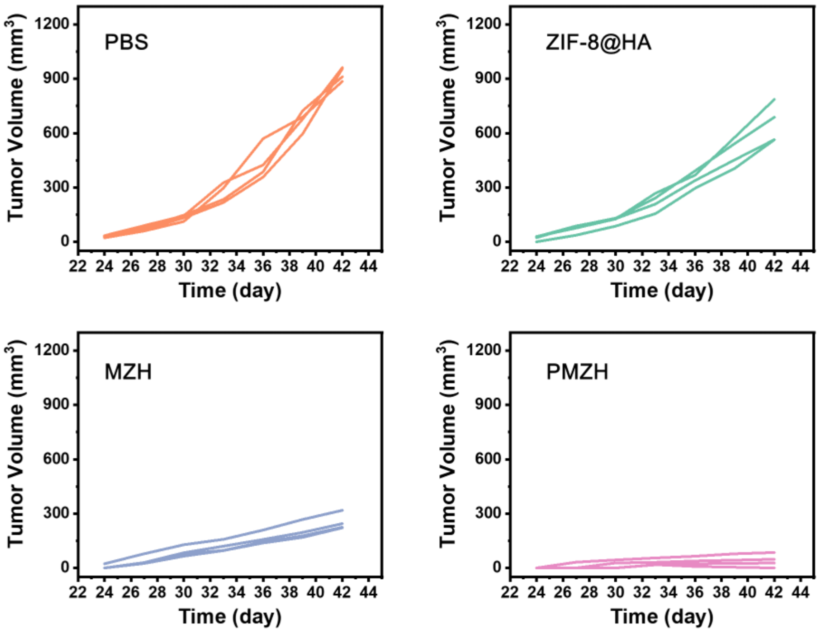

Supplementary Figure 22. Volume curves of distant tumor in PBS, ZIF-8@HA, MZH, and PMZH groups. $n = 4$ mice per group. Source data are provided as a Source Data file.

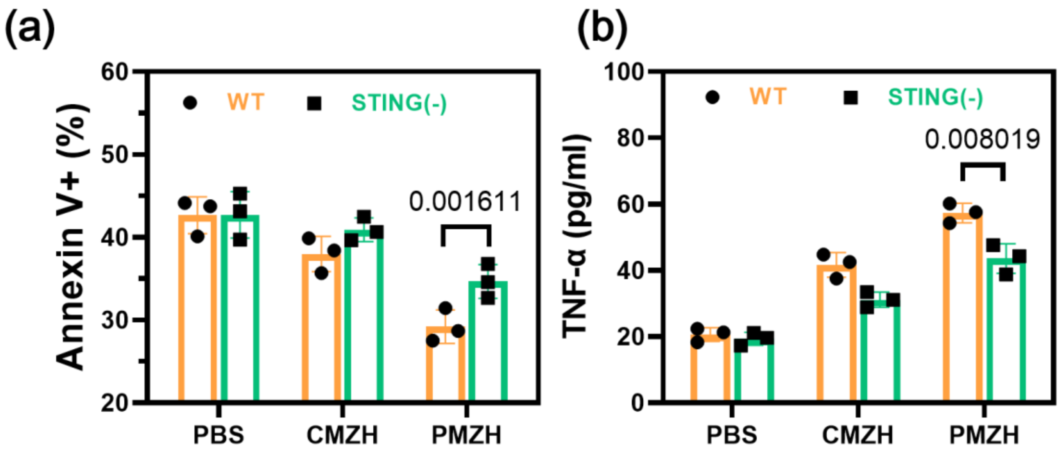

Supplementary Figure 23. (a) Effect of different treatments on apoptosis of tumor infiltrating CD8$^+$ T cells in vivo. (b) Cytokine expression levels of TNF-α in serum were analyzed by Elisa kit after different treatments. Data were presented as mean ± SD ($n = 3$ independent experiments per group in figure a and b). *P* values were assessed using Student's *t* test (two-tailed). Source data are provided as a Source Data file.

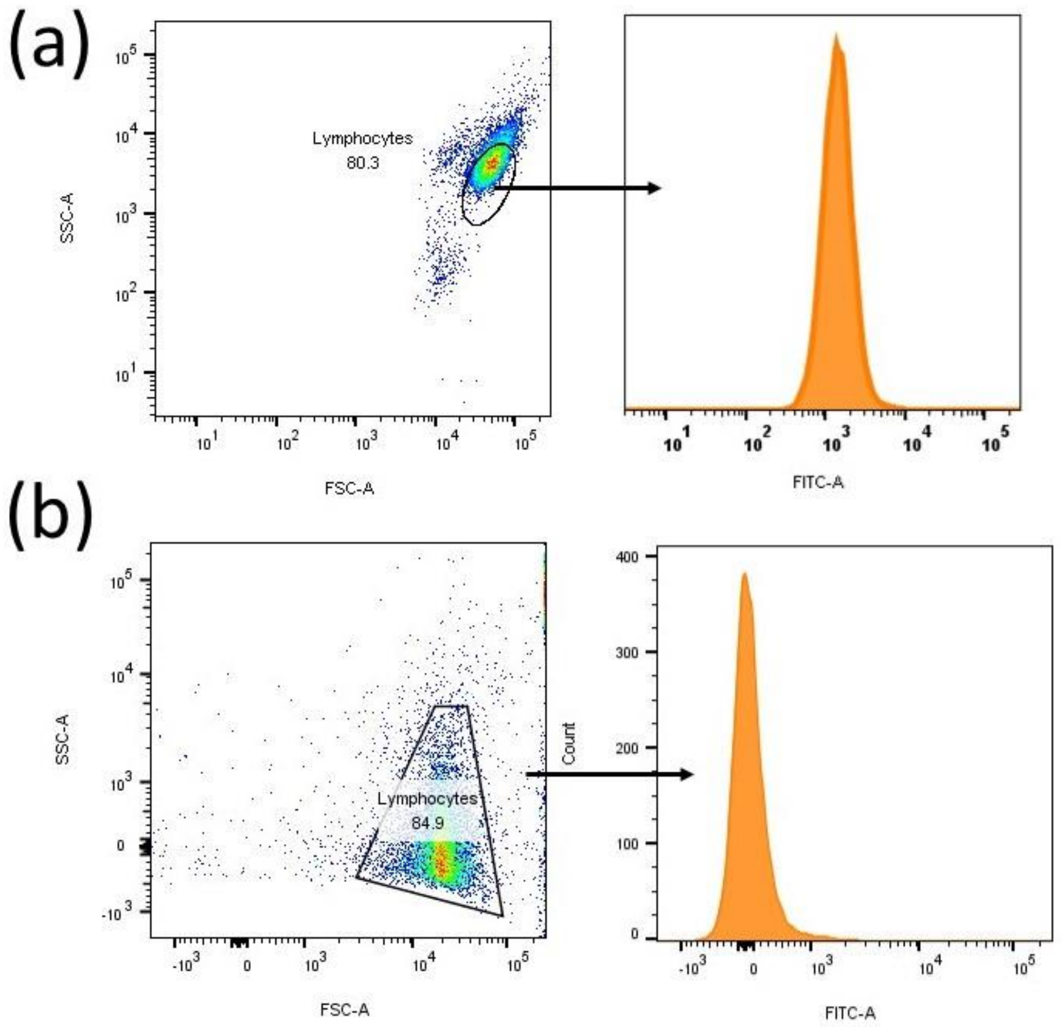

Supplementary Figure 24. Flow cytometry gating strategies for experiments in (a) Figures 3 c , Supplementary Figure 6, (b) Figures 3 f.

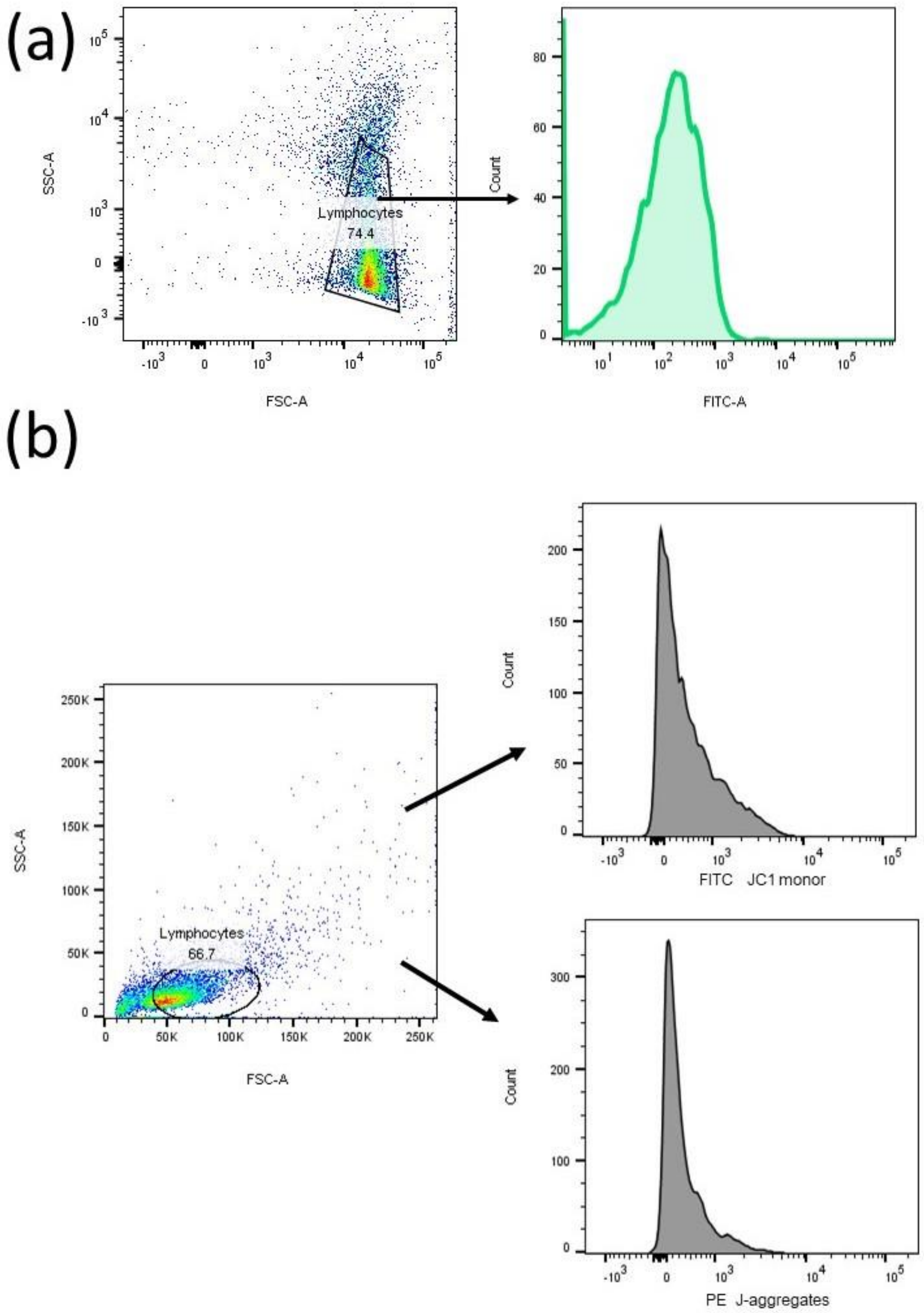

Supplementary Figure 25. Flow cytometry gating strategies for experiments in (a) Figures 5a, (b) Figures 5b.

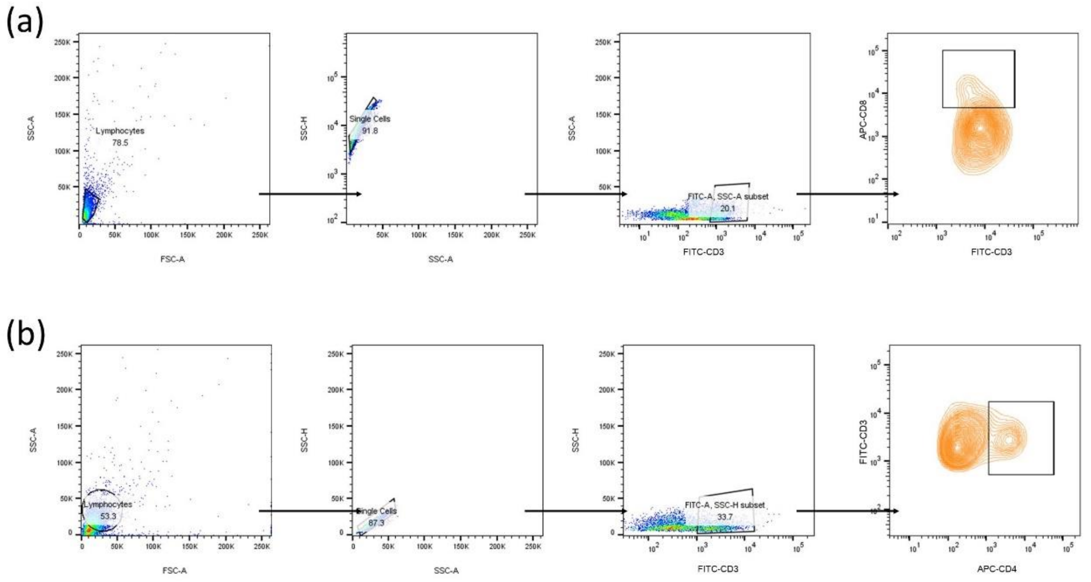

Supplementary Figure 26. Flow cytometry gating strategies for experiments in (a) Figures 7b, Supplementary Figure 19e (b) Figures 7c, Supplementary Figure 19f.
