# Supplementary material for: A bimetallic nanoplatform for STING activation and CRISPR/Cas mediated depletion of the methionine transporter in cancer cells restores anti-tumor immune responses
Source: Nat Commun. 2023 Aug 2;14:4647. doi: 10.1038/s41467-023-40345-3 (PMC10397352; doi:10.1038/s41467-023-40345-3)
Supplement: Supplementary file 3 — Reporting Summary [file 41467_2023_40345_MOESM3_ESM.pdf]

nature portfolio

Corresponding author(s): Xiaogang Qu

Last updated by author(s): Jul 6, 2023

Reporting Summary

Nature Portfolio wishes to improve the reproducibility of the work that we publish. This form provides structure for consistency and transparency in reporting. For further information on Nature Portfolio policies, see our Editorial Policies and the Editorial Policy Checklist.

Statistics

For all statistical analyses, confirm that the following items are present in the figure legend, table legend, main text, or Methods section.

n/a	Confirmed	
☐	☒	The exact sample size (*n*) for each experimental group/condition, given as a discrete number and unit of measurement
☐	☒	A statement on whether measurements were taken from distinct samples or whether the same sample was measured repeatedly
☐	☒	The statistical test(s) used AND whether they are one- or two-sided<br>*Only common tests should be described solely by name; describe more complex techniques in the Methods section.*
☐	☒	A description of all covariates tested
☒	☐	A description of any assumptions or corrections, such as tests of normality and adjustment for multiple comparisons
☐	☒	A full description of the statistical parameters including central tendency (e.g. means) or other basic estimates (e.g. regression coefficient) AND variation (e.g. standard deviation) or associated estimates of uncertainty (e.g. confidence intervals)
☐	☒	For null hypothesis testing, the test statistic (e.g. *F*, *t*, *r*) with confidence intervals, effect sizes, degrees of freedom and *P* value noted<br>*Give P values as exact values whenever suitable.*
☒	☐	For Bayesian analysis, information on the choice of priors and Markov chain Monte Carlo settings
☒	☐	For hierarchical and complex designs, identification of the appropriate level for tests and full reporting of outcomes
☐	☒	Estimates of effect sizes (e.g. Cohen's *d*, Pearson's *r*), indicating how they were calculated

Our web collection on statistics for biologists contains articles on many of the points above.

Software and code

Policy information about availability of computer code

Data collection	Transmission electron microscopy （TEM) images were acquired with a FEI TECNAI G2 20 high-resolution transmission electron microscope operating at 200 kV. The zeta potential and hydrodynamic diameters were determined by a Zetasizer 3000HS analyzer. Scanning electron microscopic (SEM) images were obtained on a Hitachi S-4800FE-SEM. Fourier-transform infrared spectroscopy (FTIR) characterization was carried out using BRUKE Vertex 70 FT-IR spectrometer. X-ray photoelectron spectroscopy (XPS) was recorded using Thermo Fisher Scientific ESCALAB 250Xi Spectrometer Electron Spectroscopy (America). ICP-MS measurements were performed on a TheroScientific Xseries inductively coupled plasmamass spectrometer. UV-Vis absorbance measurement was carried out on a JASCO V-550 UV-Vis spectrophotometer. The High Performance Liquid Chromatography (HPLC) was measured by Ultimate 3000. T1-weighted MR images were acquired using Siemens Prisma 3.0 T MR scanner (Erlangen, Germany) with gradient strength up to 80 Mt m-1.The CLSM images were acquired using a (NikonEclipse Ni-E, Japan) top-of-the-line motorized upright confocal laser scanning microscopy. The fluorescence images were recorded using an Olympus BX-51 optical equipped with a CCD camera. The protein bands were digitally captured by DNR Bio-imaging Systems (MicroChemi). The flow cytometry data was obtained by BD LSRFortessa Cell Analyzer. The whole-body fluorescence images were captured and analyzed by Tanon in vivo fluorescent imaging system and the AniView600 in vivo fluorescent imaging system.
Data analysis	Origin 2020, GraphPad Prism and Microsoft Office Excel 2019. were used for statistical analysis. FlowJo_V10. was used for analysis of Flow Cytometer. Image J sofftware was used for analysis of fluorescent images.

For manuscripts utilizing custom algorithms or software that are central to the research but not yet described in published literature, software must be made available to editors and reviewers. We strongly encourage code deposition in a community repository (e.g. GitHub). See the Nature Portfolio guidelines for submitting code & software for further information.

Data

Policy information about availability of data

All manuscripts must include a data availability statement. This statement should provide the following information, where applicable:

- Accession codes, unique identifiers, or web links for publicly available datasets
- A description of any restrictions on data availability
- For clinical datasets or third party data, please ensure that the statement adheres to our policy

All experiment data supporting the findings of this study are available within the article, Supplementary Information.

Human research participants

Policy information about studies involving human research participants and Sex and Gender in Research.

Reporting on sex and gender	This study did not involve human research participants.
Population characteristics	Not applicable.
Recruitment	Not applicable.
Ethics oversight	Not applicable.

Note that full information on the approval of the study protocol must also be provided in the manuscript.

Field-specific reporting

Please select the one below that is the best fit for your research. If you are not sure, read the appropriate sections before making your selection.

☒ Life sciences ☐ Behavioural & social sciences ☐ Ecological, evolutionary & environmental sciences

For a reference copy of the document with all sections, see nature.com/documents/nr-reporting-summary-flat.pdf

Life sciences study design

All studies must disclose on these points even when the disclosure is negative.

Sample size	All data were obtained from a minimum of 3 independent experiments and were expressed as mean ± standard deviation (SD). The statistical significance was evaluated by the student's t-test.
Data exclusions	No data were excluded from the analyses.
Replication	All experiments were conducted at least three times and could be reliably reproduced. In all experiments in which the positive and negative controls produced expected results, attempts atreplication were successful.
Randomization	The samples were divided into different groups randomly in all experiments.
Blinding	Formal blinding was used for H&E and immunofluorescence staining of tumor tissues.

Reporting for specific materials, systems and methods

We require information from authors about some types of materials, experimental systems and methods used in many studies. Here, indicate whether each material, system or method listed is relevant to your study. If you are not sure if a list item applies to your research, read the appropriate section before selecting a response.

Materials & experimental systems

n/a	Involved in the study
	☒ Antibodies
	☒ Eukaryotic cell lines
☒	☐ Palaeontology and archaeology
	☒ Animals and other organisms
☒	☐ Clinical data
☒	☐ Dual use research of concern

Methods

n/a	Involved in the study
☒	☐ ChIP-seq
	☒ Flow cytometry
☒	☐ MRI-based neuroimaging

Antibodies

Antibodies used

anti-β-actin Mouse/Rabbit 1:1000 12h Bioss N/A
anti-TBK1 Rabbit 1:1000 12 h Beyotime N/A
anti-Phospho-TBK1/NAK (Ser172) Rabbit 1:1000 12 h Beyotime N/A
anti-IFN-Beta Rabbit 1:1000/1:500 12 h Bioss N/A
anti-SLC43A2 Rabbit 1:1000 12 h Bioss N/A
anti-TMEM173/STING Rabbit 1:500 12 h Proteintech N/A
anti-CD3 Mouse 1:200 12h Biolegend N/A
anti-CD28 Mouse 1:200 12h Biolegend N/A
anti-CD11c Mouse 1:16 30 min Biolegend APC
anti-CD80 Mouse 1:30 30 min Biolegend FITC
anti-CD86 Mouse 1:30 30 min Biolegend PE
anti-CD8 Mouse 1:16 30 min Biolegend PE
anti-CD3 Mouse 1:50 30 min Biolegend FITC
anti-CD4 Mouse 1:16 30 min Biolegend APC
anti-CD8 Mouse 1:16 30 min Biolegend APC
anti-CD45 Mouse 1:16 30 min Biolegend perCP/Cyanine5.5
anti-F4/80 Mouse 1:16 30 min Biolegend FITC
anti-CD206 Mouse 1:16 30 min Biolegend perCP/Cyanine5.5
HRP-labeled Goat anti Mouse/Rabbit IgG(H+L) 1:500 30 min Bioss
FITC-labeled Goat anti Rabbit IgG (H+L) 1:500 30 min Beyotime

Validation

All antibodies used are commercially available and were validated with cells and tumor tissues for western blotting, flow cytometry and immunofluorescence staining application by the manufacturers. A validation statement for each antibody is available on the manufacturer's website.
Bioss (http://www.bioss.com.cn/)
Beyotime (https://www.beyotime.com)
Proteintech (https://www.ptgcn.com/)
Biolegend (https://www.biolegend.com/)

Eukaryotic cell lines

Policy information about cell lines and Sex and Gender in Research

Cell line source(s)

4T1 cell line (Catalog no. CRL-2539) and CT26 cell line (Catalog no. CRL-2638) were purchased from and authenticated by ATCC.

Authentication

Cell lines used were authenticated by ATCC.

Mycoplasma contamination

These cell lines were tested negative for mycoplasma contamination by the suppliers.

Commonly misidentified lines
(See ICLAC register)

None of these cell lines were used.

Animals and other research organisms

Policy information about studies involving animals; ARRIVE guidelines recommended for reporting animal research, and Sex and Gender in Research

Laboratory animals

Female BALB/c mice (22-27 g) were used in the experiment. All mice were housed in a specific pathogen-free environment at 26 ± 1 °C and 50 ± 5% humidity, with a 12 h light-dark cycle.

Wild animals

The study did not involve wild animals.

Reporting on sex

Experimental animals were mixed-gender, age-matched, and randomly assigned to into different treatment groups.

Field-collected samples	The study did not involve samples collected from the field.
Ethics oversight	All animal handling procedures were in accordance with the guidelines of the Animal Ethics Committee of Jilin University for Animal Experiments.

Note that full information on the approval of the study protocol must also be provided in the manuscript.

Flow Cytometry

Plots

Confirm that:

- [ ] The axis labels state the marker and fluorochrome used (e.g. CD4-FITC).
- [x] The axis scales are clearly visible. Include numbers along axes only for bottom left plot of group (a 'group' is an analysis of identical markers).
- [x] All plots are contour plots with outliers or pseudocolor plots.
- [x] A numerical value for number of cells or percentage (with statistics) is provided.

Methodology

Sample preparation	The 4T1 cells were seeded on a 6-well plate and grown to 80% confluency. Then the cells were incubated with FITC-labeled PMZH for 0.5,1,2,4h. They were washed with PBS, digested and analyzed by flow cytometry.<br><br>The 4T1 cells were seeded on a 24-well plate and grown to 80% confluency. Then the cells were incubated with MZH@EGFP and lipo@EGFP for 6 hours. After washing with PBS, the cells were incubated for 24 hours, and then digested and collected for analysis by flow cytometry.<br><br>4T1 cells were inoculated on a 6-well plate and cultured for 24 hours.Then the cells were incubated with different samples for 8 hours. After washing with PBS and incubation with a DCFH-DA solution (1 μM) for 1 h. All groups of cells were collected and analyzed by flow cytometry.<br><br>Mouse lymphocytes were isolated from spleen by Mouse Lymphocyte Separation Medium. CD8+ T cells were re-suspended (106 cells/ml) and activated with anti-CD3 and anti-CD28 mAbs for 48 h. Activated CD8+ T cells were maintained with IL-2 and 2-mercaptoethanol, and cultured with fresh complete medium.4T1 cells with different treatments and CD8+ T cells were cultured for 72 h in a Transwell system,and then evaluate the viability of CD8+ T cells by flow cytometer.<br><br>After therapy, the mice were sacrificed, and the tumor tissues were obtained and grinded and filtered to obtain single-cell suspension. The cell suspension was then filtered through a 70 μ m nylon filter, treated with Red Blood Cell Lysis Buffer (Solarbio), centrifuged at 450 g for 5 minutes, and washed three times with PBS containing 5% FBS. After incubation with anti-CD11c-APC, anti-CD80-FITC and anti-CD86-PE antibodies at 4 ° C for 30 minutes, the proportion of mature dendritic cells (DC) in lymph nodes was analyzed by flow cytometry. The proportion of tumor infiltrating CD4+and CD8+T cells was also determined by anti-CD8-PE, anti-CD3-FITC and anti-CD4-APC staining and subsequent flow cytometry analysis.The apoptosis of tumor cells and T cells were also determined by anti-CD45-perCP/Cyanine5.5, anti-CD8-APC and Annexin V-FITC Apoptosis Detection Kit(Beijing T&L Biological Technology Co., Ltd), staining and subsequent flow cytometry analysis.
Instrument	BD LSRFortessa Cell Analyzer
Software	BD LSRFortessa Cell Analyzer and FlowJo_V10.
Cell population abundance	The absolute cells around 10000 were analyzed for each group.
Gating strategy	Initial cell populations were gated for a live population using FSC and SSC plot of cell only sample. The gate was set to remove cell debris and dead cells.

- [x] Tick this box to confirm that a figure exemplifying the gating strategy is provided in the Supplementary Information.
